# Pancreaticobiliary maljunction with pancreas divisum: a rare variant and cause of biliary cannulation failure

**DOI:** 10.1016/j.igie.2026.01.009

**Published:** 2026-01-27

**Authors:** Ahmed Kayal

**Affiliations:** Department of Medicine, King Abdulaziz University, Rabigh Branch, Jeddah, Saudi Arabia

A previously healthy 36-year-old woman without a history of surgery, including cholecystectomy, presented to the gastroenterology clinic with mild abdominal discomfort, nausea, and fullness. She denied severe pain, vomiting, or jaundice. She was not taking any medications, including opiates and antipsychotics. Liver profile and lipase were normal. Ultrasound of the abdomen showed a markedly dilated biliary system, so magnetic resonance cholangiopancreatography (MRCP) followed. The bile duct was dilated to 1.8 cm with a “probable” stricture distally. Endoscopic retrograde cholangiopancreatography was performed next. The sphincterotome did advance slightly into the papilla, but the wire would not pass, and contrast injection failed to show any ductal structure on fluoroscopy. Before termination of the procedure, although air was being aspirated and the scope withdrawn, bile was observed draining from the minor papilla (Fig. 1).

**Figure 1:**
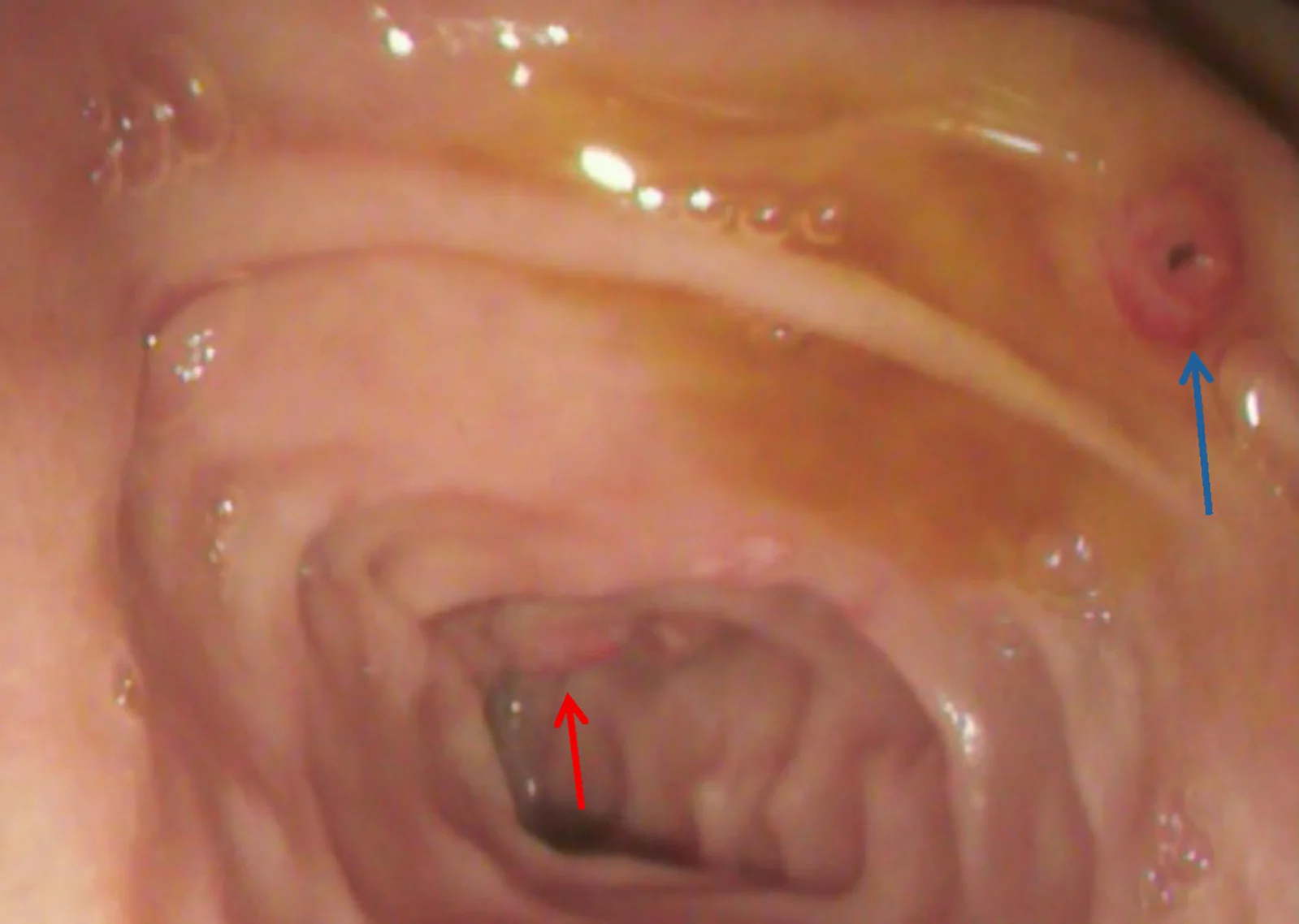
Bile from minor papilla. *Blue arrow*: minor papilla with bile draining. *Red arrow*: major papilla appears in the distance.

Upon review of the MRCP with an abdominal imaging specialist, pancreas divisum and communication of the biliary duct and dorsal duct of the pancreas were noted (Fig. 2A). In addition, the biliary duct did not appear to reach the duodenal wall (Fig. 2B), confirming a rare variant of pancreaticobiliary maljunction (PBM) (Fig. 3).^1^ The measured common channel/dorsal duct of the pancreas before communicating with the biliary duct was estimated to be 17 mm.

**Figure 2:**
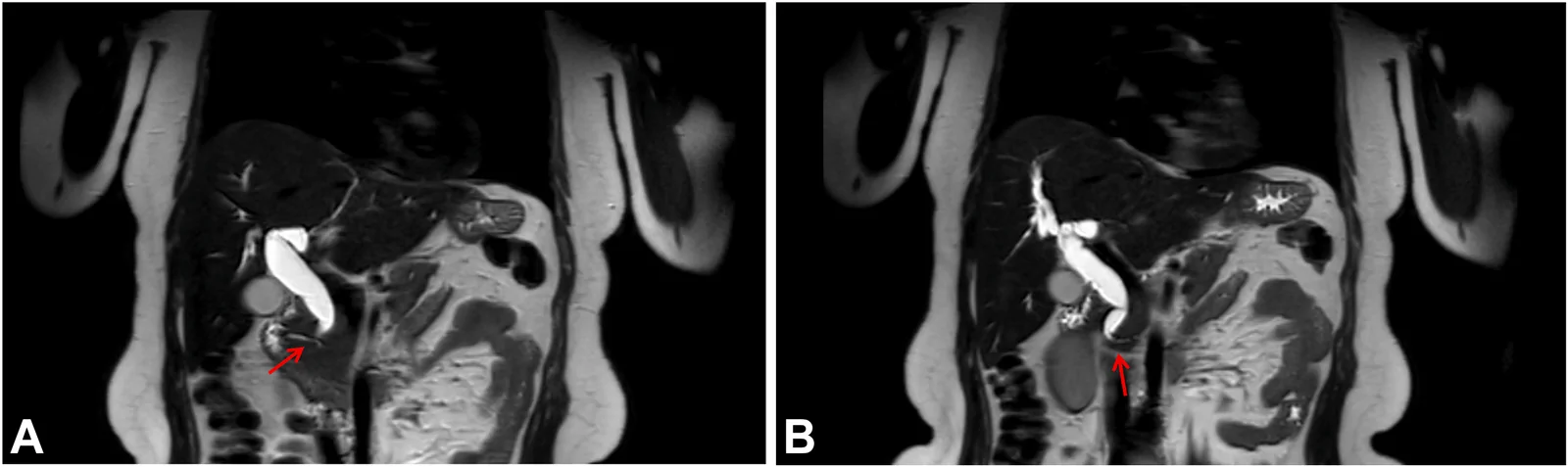
Magnetic resonance cholangiopancreatography. **A,***Arrow*: dorsal duct of pancreas/common channel measured at 17 mm. **B,***Arrow*: blind end of bile duct. The duct seems to taper without reaching the duodenal wall.

**Figure 3:**
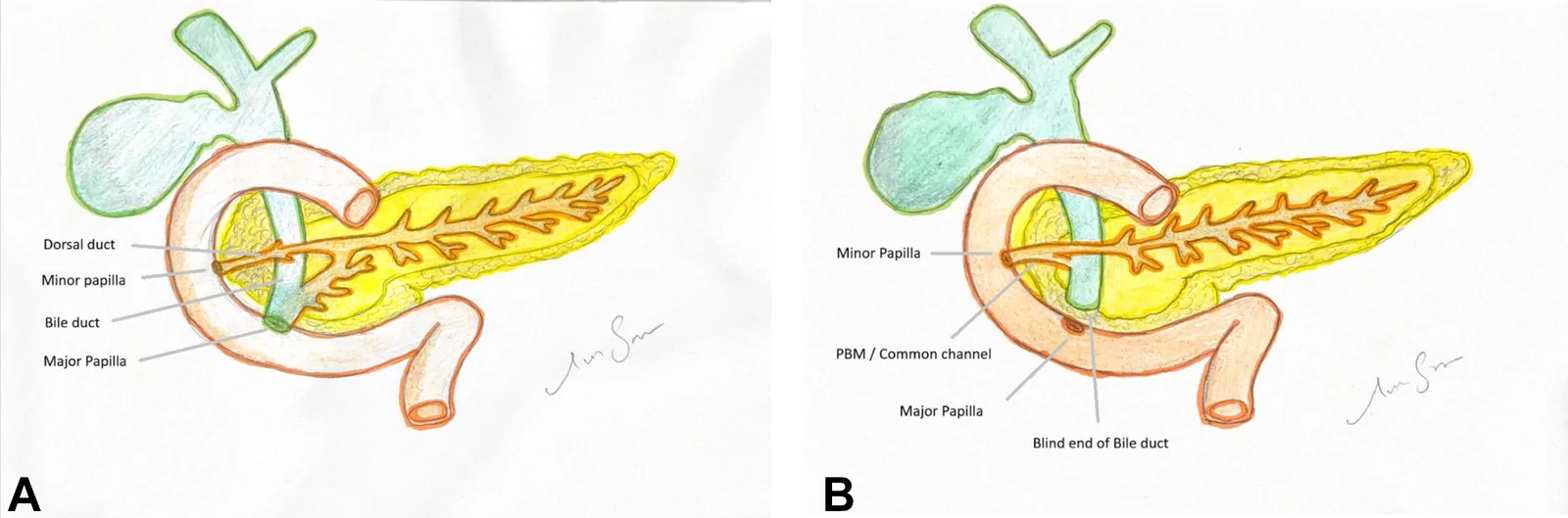
Normal and pancreaticobiliary maljunction anatomy. **A,** Normal pancreas anatomy/partial divisum. **B,** Pancreaticobiliary maljunction (PBM) with complete divisum. Common bile duct draining through the connection with the minor papilla. Courtesy of Dr Abdullah Sheitt.

Surgical resection was offered to decrease the associated malignancy risk with this condition, but the patient preferred conservative follow-up. She remained clinically stable 9 months later with a normal biochemical profile and unchanged biliary system on MRCP. We plan to continue to follow up with the patient and offer surgery again if she agrees. Endoscopic ultrasound–guided drainage is not indicated because of normal biochemical profile, minimal symptoms, and the fact that it would not decrease the associated malignancy risk of this condition. In addition, if the patient agrees to surgery, we can consider the amylase level in the bile to support the diagnosis.

PBM is a rare congenital anomaly in which the bile and pancreatic ducts join outside the duodenal wall and form a long common channel (>9 mm).^2^ An extremely rare variant of PBM divisum is presented, where both ducts drain into the minor papilla (Fig. 3B). To our knowledge, very few cases have been reported in the literature.^1^

Some reports suggest PBM is linked to recurrent acute or chronic pancreatitis, cholelithiasis, and increased risk of biliary and pancreatic malignancies.^3^ The usual treatment is surgery for this rare condition.

## Patient consent

Institutional Review Board approval was not necessary, as per the institution. All images are anonymized, and consent was obtained from the patient.

## Disclosure

The author disclosed no financial relationships.
